# The promotion of academic medicine through student-led initiatives

**DOI:** 10.5116/ijme.563a.5e29

**Published:** 2015-11-21

**Authors:** Garth Funston

**Affiliations:** 1 Department of Primary Care, Institute of Population Health, University of Manchester, Oxford Road, Manchester, UK

**Keywords:** Promotion of academic medicine, student-led initiatives

## Introduction

The decline in numbers of medically trained doctors performing research in recent decades has been widely discussed in the literature.^1^^-^^4^ The importance of engaging medical students with research and inspiring them to pursue academic careers is recognized as key in reversing the decline.^4^^-^^7^ Many medical schools offer opportunities for research participation, either as elective or compulsory components. This has been shown to increase interest in research and academic careers.^8^ However, the primary focus of curricula is the production of safe clinicians and it can be a challenge to provide adequate exposure to all aspects of academic medicine within the medical school timetable. One multi-institutional study found that while 83% of responding students felt that research participation is of educational value, only 31% felt there was adequate time set aside, 15% felt there was sufficient training in methodology and 25% believed that there was adequate training in critical appraisal.^9^ A number of extracurricular initiatives have been developed to provide additional exposure to aspects of academic medicine. Some of the most innovative and successful initiatives have been developed, and are led, by students. While student led initiatives are not a new phenomenon we believe that they have great potential to engage and inspire. Here, we discuss four of the most common student led approaches taken to promote academic medicine.

### Student research societies

Many researchers join professional associations. Student-led research societies may play a similar role, facilitating networking with other interested students and researchers, thereby helping students to feel part of the research community, and providing students with opportunities to acquire knowledge and develop relevant skills through organised events. Events organised by student societies vary, but they commonly include talks from inspiring academics, research symposia, careers talks and workshops.^10^ Attendance at such student led events has been shown to increase interest in research and encourage attendees to consider research orientated careers.^11^Some societies have taken more innovative steps such as linking students with mentors or research projects,^12^ coordinating collaborative national research studies^13^ or educating high school students about medical research.^14^Student research societies have existed at some universities for many years,  but their numbers appear to be increasing. In 2010 there were 5 medical student research societies at UK universities, while in 2014 that number had risen to 32.^10^ A google search reveals the existence of student-led research societies at medical schools in North America, Latin and South America, Australasia, Europe, Africa and Asia. In addition, national student research societies have been established in a number of countries including the UK, Pakistan, Chile and Venezuela.^4^^, ^^15^^-^^16^

### Student journals

The ability to communicate research findings via academic writing is an essential aspect of academia. While 30% of students undertaking research projects will publish in a conventional journal, eight medical student journals provide an additional outlet for academic work. Some student journals, such as the University of Toronto Medical Journal, have a long history, while a number of new journals have been launched in recent years. Although the total number of such journals is unknown,^8^ have been identified in Canada, publishing over 200 articles per year^17^ and 20 have been identified in Latin America.^18^ A google search reveals many medical student journals in Asia, Africa, Australasia, North America, South America and Europe. While some journals are small medical school specific publications, others, such as the McGill Journal of Medicine, attract international submissions and have been indexed in Medline.

To date, there has been no large systematic assessment of the quality of articles published in student journals. However, one study which looked at a specific measure of scientific impact, the H index, identified eight peer reviewed Latin American student publications with an H index of two or more and one with an H index of eleven.^18^

While it is likely that the level of quality control imposed will differ between student journals, they offer a number of potential benefits. Although a student may not have generated a sufficient quality or quantity of data to publish in a conventional journal, submitting to a student journal will provide experience of scientific writing, peer review and the publishing process. Some student journals include students as peer reviewers providing an excellent opportunity to develop critical appraisal skills. Medical student journals often contain articles specifically aimed at to helping students to develop research related skills and some publish letters, commentaries and reviews allowing students, without original data, opportunities to contribute. For those wishing to become involved in medical publishing in their future career, student journals offer a rare opportunity to hone editorial skills.^17^^, ^^19^

### Student research conferences

Research presentations are an important method of disseminating research findings and obtaining critical feedback from peers. While some professional conferences encourage students to attend and present their work, giving your first academic presentation to a large group of experts can be a daunting experience.

Student conferences can allow students to present their work in a less intimidating environment. These conferences are specifically designed to allow students to develop their presentation skills and to receive feedback. Conferences afford opportunities for students to network and can provide recognition for excellence in student research through the award of prizes. The conference program can be specifically tailored towards students’ needs with talks and workshops on topics of interest.

There has been little research into the benefits of student conferences, however, one study demonstrated that attending a medical student conference significantly increased the attendees’ interest in research.^11^

A google search reveals the existence of student research conferences in North America, Latin America, Europe, Asia, Australia and Africa. These conferences vary in scale from institution specific one day events to longer events attracting delegates from a number of countries.^20^

### Student journal clubs

The ability to appraise research and practice evidence based medicine is important for clinicians and academics alike. Journal clubs have long been seen by doctors and academics as a way of keeping up to date with medical advances and of achieving a greater understanding of published research. More recently, they have been advocated as a way to improve the reading habits, scientific knowledge and critical appraisal skills of doctors.^21^ A number of medical student-led journal clubs have been established with similar aims. While research on the impact of student journal clubs is limited, one study, which combined a journal club with a letter writing exercise, resulted in the publication of 26 letters by students.^22^

## Conclusions

While intra-curricular research training and compulsory, elective or vacation research projects should continue to form the mainstay of medical student academic training, student-led initiatives may offer a useful adjunct. They are established and coordinated by interested students, who gain experience in leadership, teamwork and organisation, thus there are no salaries which limits the cost. The students that set-up and run these initiatives are ideally placed to understand what their peers want out of events or publications.

While there is limited empirical evidence on the effects of student-led initiatives in academic medicine, their prevalence and popularity can be taken as an indication that students perceive a need for them and derive some benefit from their activities. Given the need to recruit academic clinicians it is important that further research is undertaken to determine the benefits and limitations of student led initiatives in educating students about research and promoting academic medicine.

### Conflict of Interest

The author declares that there are no conflicts of interest.
